# Optimization of Juice- and Poultry-Industry By-Product Incorporation in Cooked Sausage Formulations Using Response Surface Methodology

**DOI:** 10.3390/foods15183212

**Published:** 2026-09-11

**Authors:** Aigerim Koishybayeva, Yasin Uzakov, Shynar Kenenbay, Madina Kozhakhiyeva, Malgorzata Korzeniowska

**Affiliations:** 1 Department of Food Technology, Almaty Technological University, 050012 Almaty, Kazakhstan; aigerim.koishybayeva@atu.edu.kz (A.K.); ya.uzakov@atu.edu.kz (Y.U.); m.kozhahieva@atu.edu.kz (M.K.); 2 Department of Functional Food Products Development, Wroclaw University of Environmental and Life Sciences, 50-375 Wroclaw, Poland; malgorzata.korzeniowska@upwr.edu.pl

**Keywords:** optimization, response surface methodology, apple pomace, turkey skin, cooked sausage

## Abstract

Sustainability concerns are driving greater interest in valorizing agri-food by-products as ingredients in meat products. This study aimed to optimize a cooked turkey sausage formulation incorporating apple pomace powder (APP), turkey skin (TS), and iced water (IW) and to evaluate their effects on physicochemical, textural, and sensory properties. Response surface methodology (RSM) was applied with APP (1–5%), TS (20–30%), and IW (15–30%) as independent variables. The responses evaluated were pH, cooking yield, color parameters (L*, a*, and b*), hardness, and overall acceptability. Numerical optimization identified a formulation containing 4.45% APP, 23.19% TS, and 15.73% IW, with predicted values of pH 5.63, cooking yield 87.21%, lightness (L*) 73.78, redness (a*) 4.81, yellowness (b*) 11.35, hardness 51.21 N, and overall acceptability 6.59. Experimental validation showed that the measured responses were generally consistent with the RSM predictions, although a larger deviation was observed for b*. Principal component analysis (PCA) showed that PC1 and PC2 explained 51.66% and 28.89% of the total variance, respectively, accounting for 80.55% cumulatively. PCA provided an exploratory representation of relationships among the measured responses, with PC1 primarily contrasting pH and lightness with cooking yield, redness, and yellowness, while hardness and overall acceptability were more strongly associated with PC2. Overall, the results demonstrate the feasibility of incorporating APP and TS into cooked turkey sausage and identify an optimized formulation that balances by-product incorporation with physicochemical, textural, and sensory responses.

## 1. Introduction

Approximately one-third of global food production is lost or wasted annually, with the limited utilization of nutrient-rich by-products [1,2]. The fruit-processing industry alone has been estimated to generate approximately 0.5 billion tons of solid residues and wastewater annually [3]. Apples are the most widely produced fruits worldwide [4], and their industrial processing generates an estimated 6–12 million tons of pomace annually, much of which is still directed to landfill despite containing valuable components such as pectin, phenolic compounds, and dietary fiber [5,6]. Global meat demand has also risen by about 58% in recent decades, with per capita consumption reaching 45.8 kg per year in 2023, underscoring meat’s central role in human diets alongside its considerable environmental footprint [7,8]. Poultry meat, in particular, is projected to grow substantially, driven by urbanization and demographic change, owing to its affordability and nutritional value [9,10]. However, the expansion of poultry processing has generated substantial quantities of organic by-products, including bones, cartilage, skin, feathers, feet, and heads. Despite being rich in collagen and keratin, these by-products remain largely confined to low-value applications rather than being incorporated into high-value food products [11,12]. The incorporation of plant- and animal-processing by-products into meat formulations has therefore been investigated to valorize them while modifying the technological and nutritional characteristics of processed meat products. It is now widely recognized as an essential strategy for economic, social, and environmental sustainability [13,14,15], particularly as the challenges of solid waste management continue to intensify in response to population growth and rising consumption [16]. Because meat naturally contains negligible amounts of dietary fiber, conventional processed meat products generally contribute little to dietary fiber intake unless plant-derived ingredients are incorporated. To increase overall fiber intake, dietary fiber is now incorporated into a wide range of meat products, where its addition improves water-holding capacity, emulsion stability, and texture, thereby enhancing mouthfeel, cooking yield, and shelf life [17,18,19,20,21,22]. Apple pomace is particularly promising as a food ingredient because of its high dietary fiber content and its potential to influence the nutritional and technological properties of food products [23]. Poultry skin, in contrast, is a fat- and collagen-containing poultry by-product that can contribute to the structural and textural characteristics of processed meat products [24,25].

We hypothesized that the combined effects of apple pomace powder, turkey skin, and iced water would influence the physicochemical, textural, and sensory characteristics of cooked turkey sausages and that their levels could be jointly optimized using Response Surface Methodology (RSM). Therefore, this study aimed to investigate the effects of apple pomace powder (1–5%), turkey skin (20–30%), and iced water (15–30%) on the physicochemical, textural, and sensory characteristics of cooked turkey sausages; to develop predictive models using RSM with a CCD; and to identify and experimentally evaluate the optimized formulation.

## 2. Materials and Methods

### 2.1. Raw Materials and Chemical Composition

Turkey meat and skin were purchased from a local processor (Gizewski Ltd., Wroclaw, Poland). The approximate composition of turkey meat was 74.33% moisture, 24.5% crude protein, 0.97% crude fat, and 1.02% ash. Turkey skin contained 51.65% moisture, 10.19% crude protein, 24.33% crude fat, and 0.49% ash.

Apples were procured from a local market. After washing, the cores were removed, and the pomace was extracted by juicing the fruit. The apple pomace was frozen at −80 °C and then freeze-dried using a Free Zone 12 freeze dryer (Labconco Corporation, Kansas City, MO, USA). The process lasted 3 days, maintaining a pressure of 0.04 mbar. The freeze-dried apple pomace was then ground in a colloid mill to a powder. The resulting apple pomace powder had a moisture content of 8.89%, a fat content of 2.43%, a protein content of 2.09%, an ash content of 2.1%, a dietary fiber content of 21%, L*-value of 76.55, a*-value of 7.32, b*-value of 17.71, and a pH of 5.29. Apple pomace powder was then placed in polyethylene bags and stored at 4 °C for future use.

Raw materials (turkey meat, turkey skin, and apple pomace powder) were obtained from a single production batch and used to prepare all twenty experimental sausage formulations according to the CCD. Proximate composition, dietary fiber, color, moisture, and pH of the raw materials were determined in triplicate from this batch. Moisture was determined by measuring weight loss after drying at 105 °C for 12 h in a convection oven. Crude fat was determined using the Soxhlet extraction method with a Universal Extractor E-800 (BUCHI, Essen, Germany), while crude protein was determined using the Kjeldahl method with a Kjeltec TM 2300 nitrogen analyzer (FOSS, Hilleroed, Denmark). Ash content was determined according to the AOAC Official Methods of Analysis (17th ed.) using a muffle furnace. Total dietary fiber was determined according to the AOAC Official Methods of Analysis (16th ed.). Color parameters (L*, a*, and b*) were measured using a Minolta CR-400 reflectance colorimeter calibrated with a white standard plate. The pH was determined in a homogenate prepared from 5 g of sample and 20 mL of distilled water using an Orion 3-Star pH Benchtop Meter (Thermo Fisher Scientific Inc., Waltham, MA, USA) at room temperature. All measurements were performed in triplicate.

### 2.2. Experimental Design

Response surface methodology (RSM) is a statistical, theoretical, and mathematical technique for model building to optimize the levels of independent variables [26,27]. RSM was used to optimize the levels of apple pomace powder, turkey skin, and iced water for cooked sausages. The central composite design (CCD) was generated using Design-Expert software, version 13 (Stat-Ease, Inc., Minneapolis, MN, USA). Three independent variables were investigated: apple pomace powder (X_1_), turkey skin (X_2_), and iced water (X_3_). Table 1 lists the experimental factor levels and their corresponding coded values. 20 treatments, including 8 factorial points, 6 axial points, and 6 center points, were randomly performed according to the CCD, as summarized in Table 2. The actual levels of the independent variables were coded according to Equation (1).

Xi = Zi − Z0/ΔZ(1)

where Zi is the actual value of the independent variable, Z0 is the value at the design center, and ΔZ is the step change. The specific equations for each independent variable were derived from the above equation to code their actual values. Specific equations for apple pomace powder (X_1_), turkey skin (X_2_), and iced water (X_3_) are mentioned in Equations (2)–(4) below:

X_1_ = (APP − 3)/2 (2)

X_2_ = (TS − 25)/5 (3)

X_3_ = (IW − 22.5)/7.5 (4)

where APP, TS and IW represent apple pomace powder, turkey skin and iced water, respectively.

For each CCD run, the three technical measurements of the physicochemical and instrumental responses were averaged, and the resulting mean served as the single response value for RSM analysis. Overall acceptability was calculated from hedonic ratings provided by the sensory panel. Analysis of variance (ANOVA) was used to determine the adequacy of the model based on the coefficient of determination (R^2^), adjusted R^2^, predicted R^2^, the lack of fit at the 95% confidence level, and Fisher’s test value (F-value). Model adequacy was evaluated using the model F-value and associated *p*-value, coefficient of determination (R^2^), adjusted R^2^, predicted R^2^, and the lack-of-fit test at a significance level of *p* < 0.05. Model reduction was performed using backward elimination with a significance threshold of *p* = 0.05, while preserving model hierarchy. Three-dimensional response-surface plots were generated to visualize the effects and interactions of the formulation variables, with two factors varied simultaneously while the third was held constant. Numerical optimization was performed using the desirability function. The optimization goals were to minimize pH, lightness (L*), and yellowness (b*) and to maximize cooking yield, redness (a*), hardness, and overall acceptability. The combination of apple pomace powder, turkey skin, and iced water that provided the highest overall desirability was selected as the optimized formulation.

A second-order polynomial equation was used to indicate the predicted responses (pH, cooking yield, lightness, redness, yellowness, hardness, and overall acceptability) as a function of an independent variable as follows (Equation (5)):

Z = β_0_ + β_1_X_1_ + β_2_X_2_ + β_3_X_3_ + β_11_X_1_^2^ + β_22_X_2_^2^ + β_33_X_3_^2^ + β_12_X_1_X_2_ + β_13_X_1_X_3_ + β_23_X_2_X_3_ (5)

where Z represents predicted response value, X_1_, X_2_, and X_3_ represent the independent variables (apple pomace powder, turkey skin, and iced water content, respectively); β_0_ is the intercept (constant); β_1_, β_2_, and β_3_ are the linear coefficients; β_11_, β_22_, and β_33_ are the quadratic coefficients; and β_12_, β_13_, and β_23_ are the interaction coefficients.

### 2.3. Sausage Formulation and Emulsion Preparation

Deboned turkey meat and skin, previously frozen, were thawed under controlled refrigeration at 4 °C. After thawing, the meat and skin were separately ground using an electric meat mincer (W-82AN, Spomasz, Żary, Poland) through 8-mm and 4-mm plates, respectively. Sausages were formulated according to the ingredient proportions specified for each experimental run in the CCD matrix. Each CCD formulation was produced as a single sausage-production batch; no independent production-batch replication was performed. APP was incorporated at 1–5%. TS content ranged from 20 to 30%, and IW ranged from 15 to 30%. Non-meat ingredients, such as curing salt (1.6%), isolated soy protein (1.6%), and sugar (0.5%), were kept constant across all formulations. Turkey meat was used as the balancing ingredient, and its proportion was calculated to ensure a total formulation of 100% (*w*/*w*).

The proportion of turkey meat was calculated as follows:

Turkey meat (%) = 100 − (APP + TS + IW + curing salt + isolated soy protein + sugar).

where APP, TS and IW represent apple pomace powder, turkey skin and iced water, respectively.

After adding all non-meat ingredients (curing salt, isolated soy protein, sugar) to the comminuted meat, the mixture was homogenized at 9000 rpm for 30 s using a Büchi Mixer B-400 (BÜCHI Labortechnik GmbH, Essen, Germany) to achieve a stable meat emulsion. The prepared raw emulsion was manually stuffed into 60 g polypropylene tubes. Thermal processing was performed in a temperature-controlled water bath set at 80 °C. Cooking was considered complete when the internal temperature reached 72 °C. Immediately after heat treatment, the sausages were rapidly cooled in an ice bath to room temperature.

### 2.4. The Chemical Characteristics of Cooked Sausages

#### pH Evaluation

The pH of cooked sausage samples was measured immediately after cooling to room temperature. A homogenate was prepared by blending 5 g of the sample with 20 mL of distilled water, and the pH was measured at room temperature using a calibrated pH meter (Orion 3-Star pH Benchtop Meter, Thermo Fisher Scientific Inc., Waltham, MA, USA). All measurements were performed in triplicate, and the mean values were reported.

### 2.5. The Physical Characteristics of Cooked Sausages

#### 2.5.1. Cooking Yield

Cooking yield was determined by recording the weight of each cooked sausage sample immediately before and after thermal processing on a calibrated electronic balance. Cooking yield was expressed as a percentage, calculated as the ratio of cooked weight to raw weight multiplied by 100. All measurements were performed in triplicate, and the mean values were reported.

#### 2.5.2. Color Evaluation

The instrumental color of cooked sausage samples was measured using a reflectance colorimeter (Minolta CR-400, Konica Minolta, Tokyo, Japan), calibrated against a standard white plate (L* = +97.83, a* = −0.43, b* = +1.98). Color was reported in CIE L* (lightness), a* (redness), and b* (yellowness). Before measurement, cooked sausage samples were sliced uniformly (15 × 25 mm), and measurements were taken immediately after production. All color determinations were performed in triplicate.

#### 2.5.3. Texture Profile Analysis

TPA of cooked sausages was conducted using the two-cycle compression procedure described by Bourne [28] on a Zwick/Roell Z010 universal testing machine (Zwick Testing Machines Ltd., Leominster, Herefordshire, UK). Three cylindrical slices per treatment (diameter: 25 mm; height: 15 mm) were compressed twice to 50% of their original height under the following instrumental conditions: 50% deformation, a crosshead speed of 60 mm/min, and a 30 s relaxation time between compression cycles. Hardness was defined as the maximum force recorded during the first compression cycle and expressed in N. All measurements were conducted in triplicate at room temperature (22 °C) immediately after production. Hardness was selected as the textural response in the CCD optimization because it directly measures the sausage matrix’s resistance to compression and represents product firmness.

### 2.6. The Sensory Characteristics of Cooked Sausages

The sensory attributes of the cooked turkey sausages were evaluated using a nine-point hedonic scale, ranging from 1 (dislike extremely) to 9 (like extremely) [29]. Panelists [N = 10], who were already familiar with the evaluation criteria established for this study, assessed the samples for smell, color, taste, texture, juiciness, and overall acceptability. All panelists provided verbal consent before the sensory evaluation, and participation was voluntary. No names or genders were recorded to ensure privacy. The evaluation was conducted in a well-ventilated room with neutral-colored walls, proper lighting, and no distractions, prior to the midday meal to minimize the influence of satiety on panelists’ responses. Sausage samples were cut into uniform 1 cm slices, served warm on white plastic plates, and presented to each panelist in individually coded portions. Panelists rinsed their palates with warm water between samples to prevent carryover effects. Overall acceptability was selected as the key response variable in the CCD optimization because it integrates panelists’ collective sensory perceptions.

### 2.7. Experimental Validation of the Optimized Formulation

Following numerical optimization, the selected optimized formulation was prepared under the same processing conditions described in Section 2.3. The numerical optimum values obtained from the desirability function (4.45% apple pomace powder, 23.19% turkey skin, and 15.73% iced water) were used directly, without rounding, for the preparation of the validation batch, in order to verify the accuracy of the model’s predicted responses. The physicochemical and instrumental responses were determined using the same analytical procedures applied to the CCD formulations, and the sensory response was evaluated using the same hedonic assessment procedure. The experimentally obtained response values were compared with the corresponding values predicted by the RSM models to evaluate the predictive performance of the developed models.

### 2.8. Principal Component Analysis of Cooked Sausages

Principal component analysis (PCA) was performed using OriginPro 2026b (OriginLab Corporation, Northampton, MA, USA) as an exploratory multivariate analysis of the physicochemical, textural, and sensory responses of the 20 CCD formulations. Seven response variables were included: pH, cooking yield, lightness (L*), redness (a*), yellowness (b*), hardness, and overall acceptability. Because the variables were expressed in different units and scales, PCA was performed using the correlation matrix after mean-centering and standardization to unit variance. The first two principal components were retained for graphical interpretation based on the proportion of total variance explained. The RSM-derived optimized formulation was not included in the PCA; therefore, PCA was used only to explore the multivariate response structure and not to validate the RSM optimization. For graphical interpretation, the CCD runs were additionally grouped according to APP, TS, and IW levels. For each factor, coded levels <0, =0, and >0 were classified as low, medium, and high, respectively. These groupings were used only for visualization and not to quantify factor effects or their relative importance.

## 3. Results and Discussion

### 3.1. Model Fitting of the Experimental Data

The effects of apple pomace powder, turkey skin, and iced water on the physicochemical characteristics and sensory properties of cooked sausage formulations enriched with apple pomace powder are presented in Table 3.

ANOVA was used to assess the linear and quadratic effects of apple pomace powder, turkey skin, and iced water contents on cooked sausage properties. Table 3 presents the model’s statistical significance, variables, and interactions, along with model coefficients and lack of fit. Model equations were developed using linear or quadratic terms for the dependent and independent variables. Model coefficients, *p*-values, and lack-of-fit values were used to characterize the degree of fit. Regression models effectively predicted the effects of the independent variables on the responses. A reduced quadratic model described responses including cooking yield, yellowness (b*), and hardness, whereas a reduced linear model characterized pH, lightness (L*), redness (a*), and overall acceptability.

Table 4 presents the reduced linear and reduced quadratic model equations for the variables. The regression coefficients of the second-order polynomial equations were derived from the experimental data to predict each response variable as a function of the coded independent variables. The calculated model values for each response were statistically significant at the 95% confidence level (*p* < 0.05). A smaller *p*-value and a larger F-value indicate a highly significant effect of any term on the response variable [30]. The F-values obtained with Fisher’s test showed low *p*-values for all independent variables (*p* < 0.05), except for the effects of iced water on pH (ns) and turkey skin on yellowness (ns) of cooked sausages. In this study, the lack-of-fit values for all responses were insignificant (*p* > 0.05). The R^2^ values, expressing the percent change in the model responses, varied between 0.8424 and 0.9899 (Table 3).

### 3.2. Effect of Independent Variables on Response Variables

Cooked sausages were prepared at different levels of the independent variables (Table 2). The effects of the independent variables on pH, cooking yield, lightness (L*), redness (a*), yellowness (b*), hardness, and OAA are presented in Table 2. Regression coefficients for the independent variables are summarized in Table 3.

#### 3.2.1. Effect of Variables on Physicochemical Characteristics of Cooked Sausages

##### pH Value of Cooked Sausages

**Figure 1:**
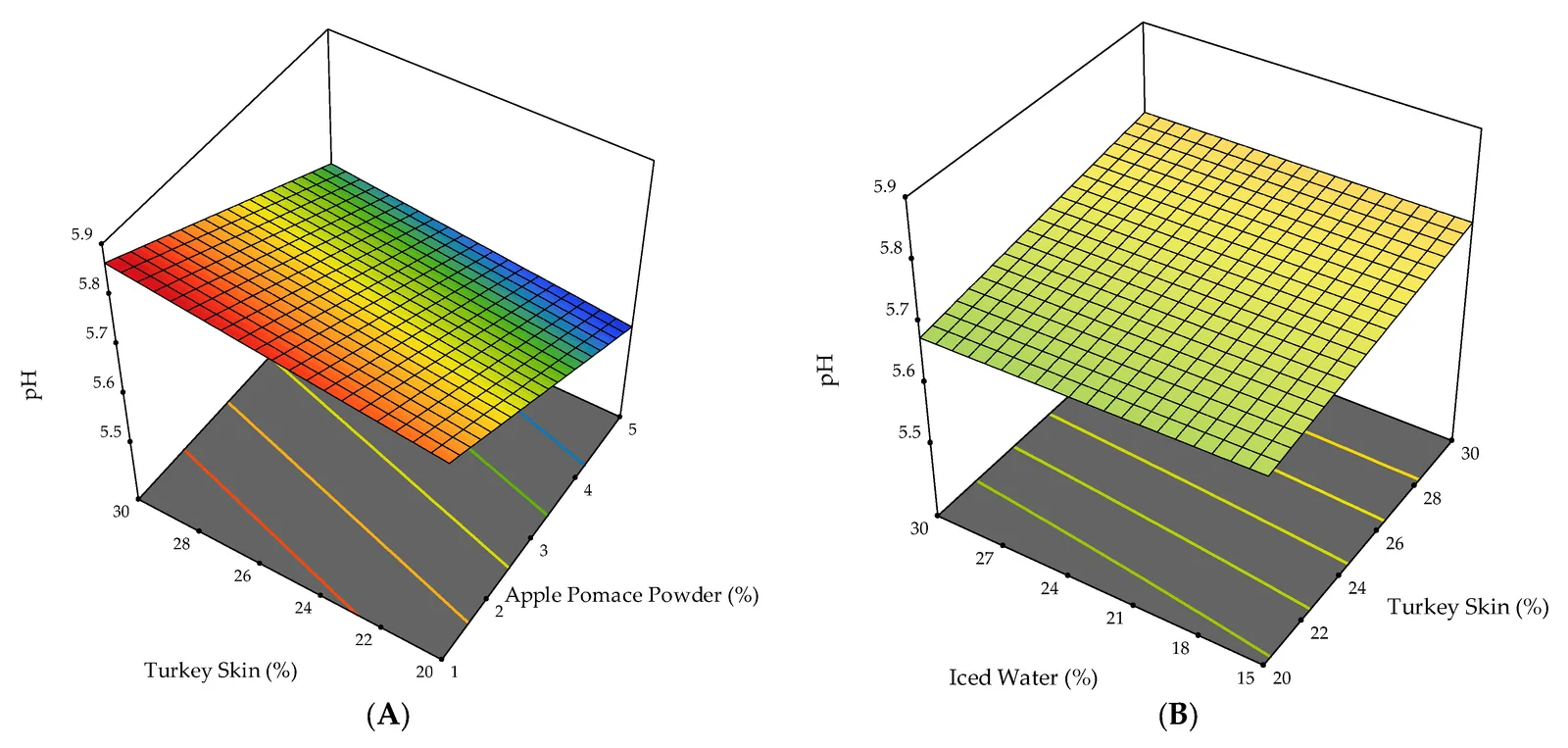

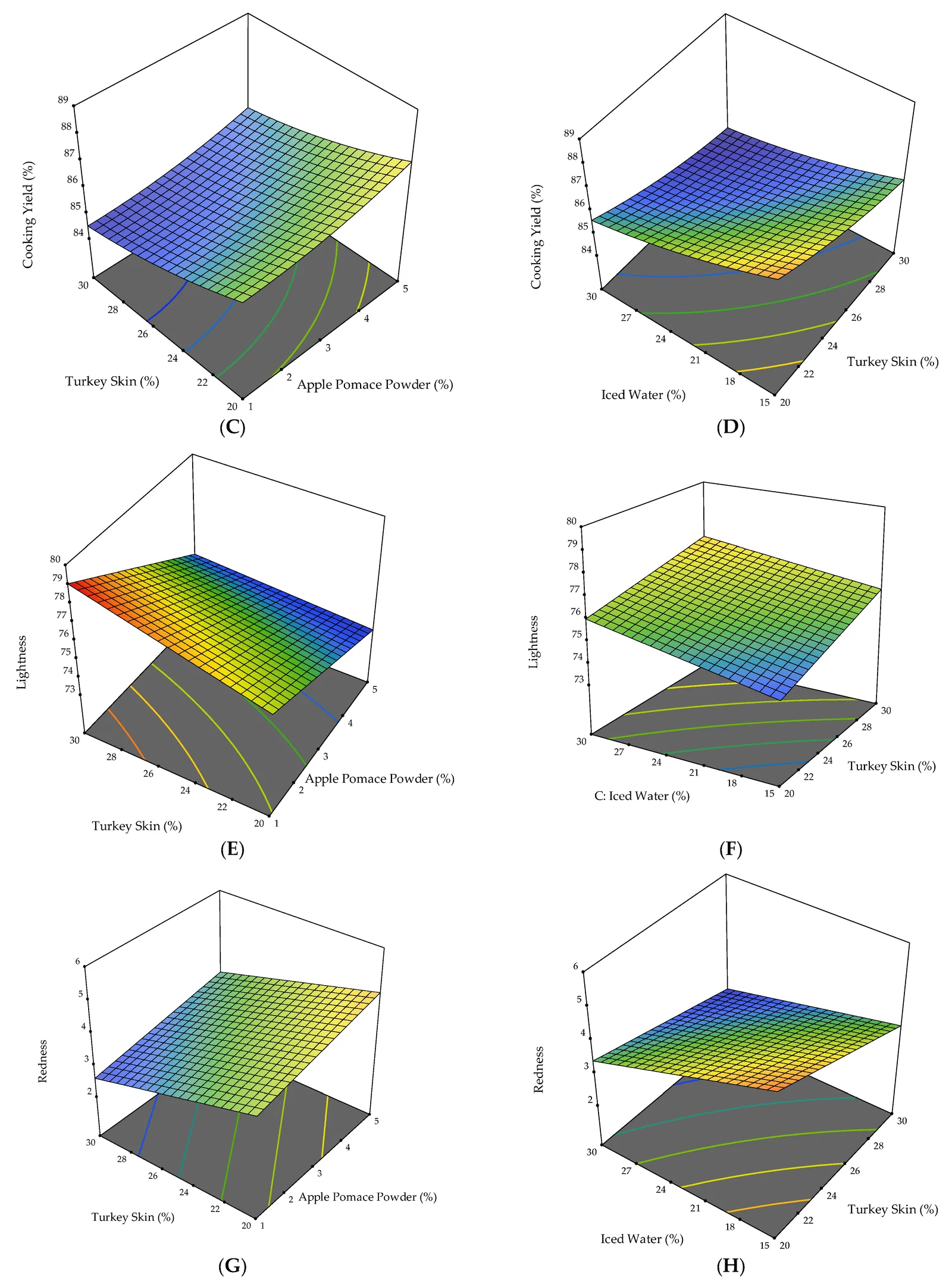

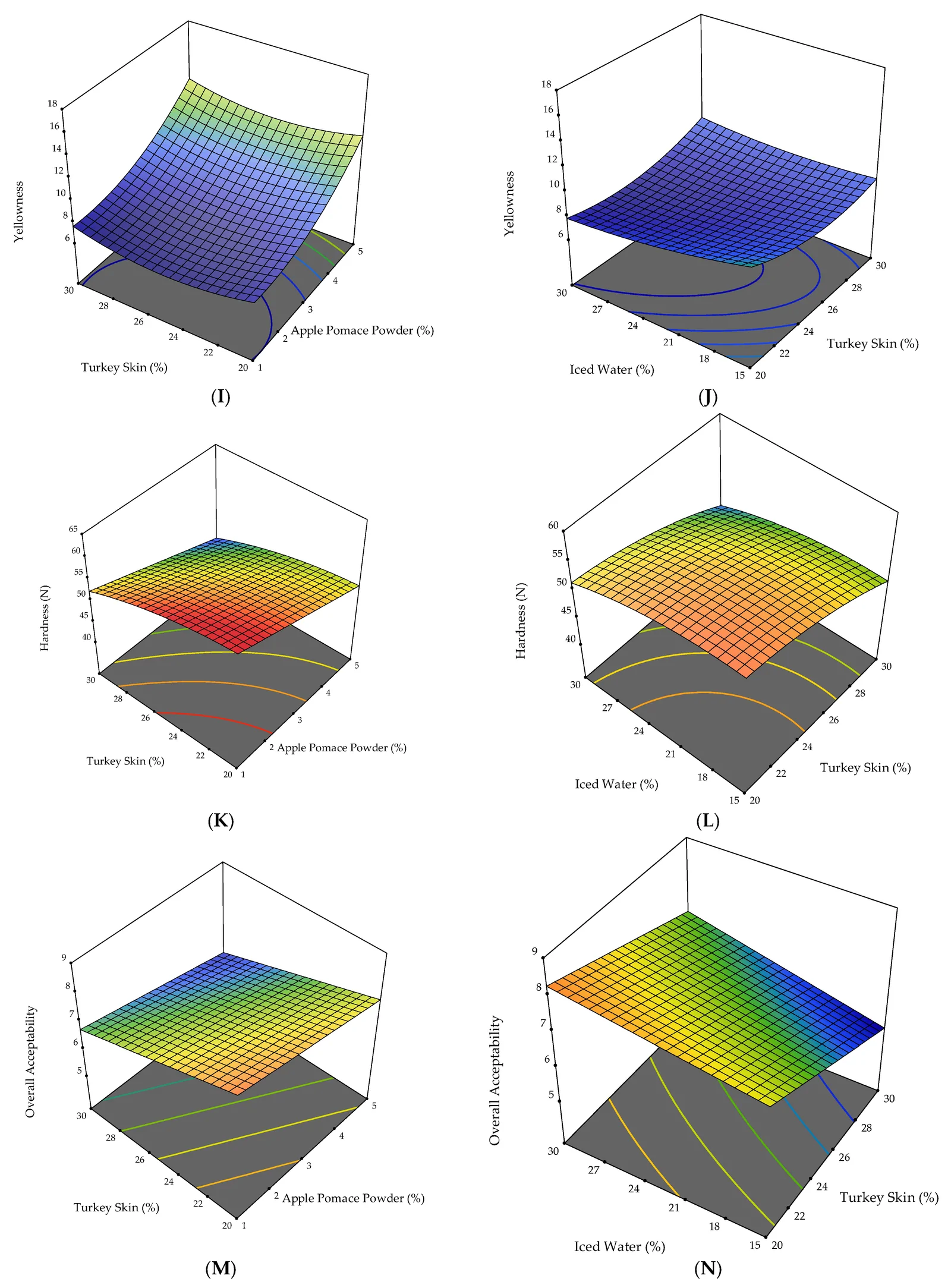
3D graphic surface optimization of (**A**) pH versus apple pomace powder and turkey skin; (**B**) pH versus turkey skin and iced water; (**C**) cooking yield (%) versus apple pomace powder and turkey skin; (**D**) cooking yield (%) versus turkey skin and iced water; (**E**) lightness versus apple pomace powder and turkey skin; (**F**) lightness versus turkey skin and iced water; (**G**) redness versus apple pomace powder and turkey skin; (**H**) redness versus turkey skin and iced water; (**I**) yellowness versus apple pomace powder and turkey skin; (**J**) yellowness versus turkey skin and iced water; (**K**) hardness (**N**) versus apple pomace powder and turkey skin; (**L**) hardness (**N**) versus turkey skin and iced water; (**M**) overall acceptability versus apple pomace powder and turkey skin; (**N**) overall acceptability versus turkey skin and iced water. The surface color gradient represents the magnitude of the response: cooler colors (e.g., blue) indicate lower response values, while warmer colors (e.g., yellow to red) correspond to higher response values.

The pH of cooked sausages depended on apple pomace powder, which had a significant linear effect on pH (*p* < 0.001). Another independent variable with a significant effect on pH was the linear effect of turkey skin content (*p* < 0.05) (Table 3). Table 3 presents the coefficients of the variables in the regression model for the pH of cooked sausages. Apple pomace powder (APP) had the strongest effect on pH (F = 88.16, *p* < 0.001, β = −0.11), with increasing APP concentration decreasing the pH of cooked sausages (Figure 1A). This effect may be related to organic acids naturally present in apple pomace, including malic acid, and is consistent with previous reports of lower pH after incorporating apple pomace into meat products [19,31,32,33,34,35,36,37,38,39]. Turkey skin had a smaller but still significant positive effect (F = 7.59, *p* < 0.05; β = +0.03), with increasing turkey skin content resulting in a slight increase in the pH of cooked sausages. Similar findings have been reported for poultry skin, which showed a higher pH than breast meat after different treatments and cooking conditions [40]. Increased pH values have also been reported in sausages and other poultry products formulated with skin [25,41]; however, the effect of poultry skin on product pH is not consistent across formulations. The combination of poultry skin and wheat fiber as a fat replacer did not significantly affect the pH of reduced-fat emulsion-type chicken sausages and nuggets [42,43], whereas the use of a pre-emulsified duck skin–pectin mixture as a fat replacer resulted in lower pH values [44]. These findings indicate that the effect of skin incorporation on product pH may depend on the type and level of skin, other formulation ingredients, and processing conditions. Iced water had no significant linear effect on pH (F = 0.04, *p* > 0.05; β = −0.003 (Figure 1B).

##### Cooking Yield of Cooked Sausages

Cooking yield is a key performance indicator for emulsion-type meat products because it integrates water- and fat-holding capacity and emulsion stability during thermal processing. All three factors had significant linear (*p* < 0.001) and quadratic (*p* < 0.05) effects on cooking yield (Table 3).

APP showed a positive linear effect (F = 22.43, *p* < 0.001; β = +0.45), whereas turkey skin (F = 81.10, *p* < 0.001; β = −0.86) and iced water (F = 98.82, *p* < 0.001; β = −0.95) showed negative linear effects (Figure 1C,D). The improvement in cooking yield with increasing APP may be associated with the water-retention properties of its dietary fiber, as previously reported for fiber-containing meat formulations [32,33,45,46,47,48]. In contrast, greater incorporation of turkey skin may reduce the proportion of myofibrillar proteins available for binding water and fat, while higher added-water levels may exceed the retention capacity of the meat matrix, increasing cooking losses [42,49,50,51,52,53]. The significant quadratic terms indicate that these effects were nonlinear across the investigated formulation space.

##### Color Properties of Cooked Sausages

Color is a primary sensory attribute that affects consumer acceptance of cooked sausages. The lightness of cooked sausages depended on apple pomace powder, which had a significant linear effect on lightness (*p* < 0.001). Other independent variables that had significant effects on the lightness of cooked sausages were the linear effects of turkey skin (*p* < 0.01) and iced water content (*p* < 0.01).

All three formulation factors significantly affected lightness (L*). APP had a negative effect (F = 64.20, *p* < 0.001; β = −1.81), whereas turkey skin (F = 12.32, *p* < 0.01; β = +0.79) and iced water (F = 9.02, *p* < 0.01; β = +0.68) increased L* values (Figure 1E,F). The decrease in lightness with increasing APP may be associated with the intrinsic color of the pomace and other naturally occurring colored constituents. Similar decreases in L* following apple pomace incorporation have been reported in several meat products [19,33,39,54,55]. The higher L* values observed with turkey skin and iced water may reflect dilution of muscle pigments and changes in light scattering within the meat matrix [56,57,58].

The redness of cooked sausages depended on all three factors, and each had a significant linear effect (*p* < 0.001). Redness (a*) was also significantly influenced by all three factors. APP increased redness (F = 27.22, *p* < 0.001; β = +0.38), while turkey skin (F = 78.02, *p* < 0.001; β = −0.65) and iced water (F = 97.29, *p* < 0.001; β = −0.73) decreased it (Figure 1G,H). The increase associated with APP is consistent with previous studies reporting darker and more reddish meat products after apple-pomace incorporation [17,33,38,39,59,60,61]. Conversely, replacing lean meat with skin or adding water can reduce the concentration of muscle pigments in the formulation, contributing to lower a* values [19,56,57,62,63].

The yellowness of cooked sausages depended on apple pomace powder and iced water, with a significant linear effect (*p* < 0.001), a quadratic effect of apple pomace powder, turkey skin (*p* < 0.001), and iced water (*p* < 0.01), and an interaction between turkey skin and iced water (*p* < 0.001). APP had a strong positive linear effect (F = 679.22, *p* < 0.001; β = +2.52), whereas iced water had a significant negative effect (F = 33.53, *p* < 0.001; β = −0.56). The linear effect of turkey skin was not significant (F = 0.16, *p* > 0.05), although significant quadratic effects and a turkey skin × iced water interaction were observed (Table 3). The increase in b* with APP is consistent with previous studies reporting increased yellowness after incorporating apple pomace into meat products [31,32,34,35,59]. The decrease associated with iced water may reflect dilution of color-contributing components within the formulation. The significant nonlinear and interaction effects indicate that the influence of turkey skin on yellowness depended on its level and the amount of iced water rather than on a simple linear relationship.

##### Hardness of Cooked Sausages

The hardness of cooked sausages depended on the apple pomace powder and turkey skin, which had a significant linear effect (*p* < 0.001) and iced water (*p* < 0.01), and a quadratic effect of turkey skin and iced water (*p* < 0.05).

APP, turkey skin, and iced water significantly affected hardness. APP showed the largest negative linear effect (F = 64.40, *p* < 0.001; β = −4.63), followed by turkey skin (F = 38.64, *p* < 0.001; β = −3.58) and iced water (F = 13.21, *p* < 0.01; β = −2.10) (Figure 1K,L). Thus, increasing each component tended to produce a softer sausage matrix. Previous studies have shown that the effect of apple-pomace fiber on texture varies with fiber content, particle size, and interactions with muscle proteins, with both decreases and increases in hardness reported [19,32,34,59,61,64,65,66]. Higher skin and water levels may also reduce matrix firmness by decreasing the relative concentration of structural meat proteins and altering gel formation [49,50,67]. Significant quadratic effects of turkey skin and iced water further indicate nonlinear effects on texture.

#### 3.2.2. Effects of Variables on Sensory Properties of Cooked Sausages

The overall acceptability of cooked sausages depended on all three factors, each of which had a significant linear effect (*p* < 0.001).

All three formulation factors significantly affected overall acceptability (*p* < 0.001). APP had a negative effect (F = 18.25; β = −0.46), with acceptability decreasing as APP concentration increased (Figure 1M). Previous studies similarly indicate that moderate APP incorporation can be acceptable, whereas higher concentrations may reduce sensory scores because of changes in color, flavor, and texture [19,33,35,38,46,61]. Turkey skin had the largest negative effect on overall acceptability (F = 57.28, *p* < 0.001; β = −0.81). Increasing turkey skin reduced the hedonic scores assigned by the study panel, which may reflect changes in mouthfeel and texture at higher skin levels. Previous studies have similarly shown that the sensory effects of poultry skin depend strongly on incorporation level and formulation, with moderate concentrations sometimes producing acceptable scores but excessive inclusion reducing sensory quality [24,25,50,51,68]. In contrast, iced water had a significant positive effect on overall acceptability (F = 22.82, *p* < 0.001; β = +0.51) (Figure 1N). Within the investigated range, increasing iced water was associated with higher panel scores, particularly at lower turkey skin levels. This effect may be related to the contribution of added water to product tenderness and juiciness, as similar relationships between water level, texture, and sensory scores have been reported for emulsion-type sausages [52].

### 3.3. Optimization of Independent Variables

To illustrate the effects of apple pomace powder, turkey skin and iced water on the response variables, response surface graphs were generated using Design-Expert Software. These graphs were produced by varying two independent variables within the experimental ranges while holding the third at its central value. Figure 1A,C,E,G,I,K,M were generated by varying the apple pomace powder and turkey skin content at a central value of iced water content (22.5%), while Figure 1B,D,F,H,J,L,N were drawn by varying the content of turkey skin and iced water at a central value of apple pomace powder concentration (3%). These graphs illustrated the predicted response trends across the investigated formulation space.

After that, numerical optimization was performed using the desirability function in Design Expert Software. The goals selected for optimizing cooked sausages were to maximize the levels of apple pomace powder and turkey skin, within a range of iced water levels to obtain a lower pH, higher cooking yield, lower lightness and yellowness, and higher redness, and to maximize hardness and overall acceptability. The study aimed to promote a firmer product structure within the observed experimental range. Three solutions were found, each with different levels of the independent variables. The solution with the maximum desirability value (0.60) was selected as the optimized cooked sausage condition. The combined optimized preparation conditions for cooked sausages were 4.45% apple pomace powder, 23.19% turkey skin, and 15.73% iced water. The response values at the optimized preparation conditions were 5.63 pH, 87.21% cooking yield, 73.78 lightness, 4.81 redness, 11.35 yellowness, 51.21 N hardness, and 6.59 overall acceptability (Table 5).

### 3.4. Experimental Validation of the RSM Models

The optimized formulation was experimentally evaluated to assess the predictive performance of the RSM models. The formulation containing 4.45% APP, 23.19% TS, and 15.73% IW was prepared under the optimized conditions, and the experimentally determined responses were compared with the corresponding model-predicted values (Table 5). The predicted values were pH 5.63, cooking yield 87.21%, lightness (L*) 73.78, redness (a*) 4.81, yellowness (b*) 11.35, hardness 51.21 N, and overall acceptability 6.59. The corresponding experimental values were pH 5.58 ± 0.04, cooking yield 87.89 ± 0.27%, lightness (L*) 73.75 ± 0.45, redness (a*) 4.90 ± 0.06, yellowness (b*) 13.49 ± 0.99, hardness 53.36 ± 0.53 N, and overall acceptability 6.97 ± 0.12. The experimental values were generally consistent with the model predictions, supporting the predictive performance of the developed RSM models within the investigated formulation space. A comparatively larger deviation was observed for b*, indicating lower predictive agreement for yellowness than for the other responses.

### 3.5. Principal Component Analysis of Cooked Sausages

**Figure 2:**
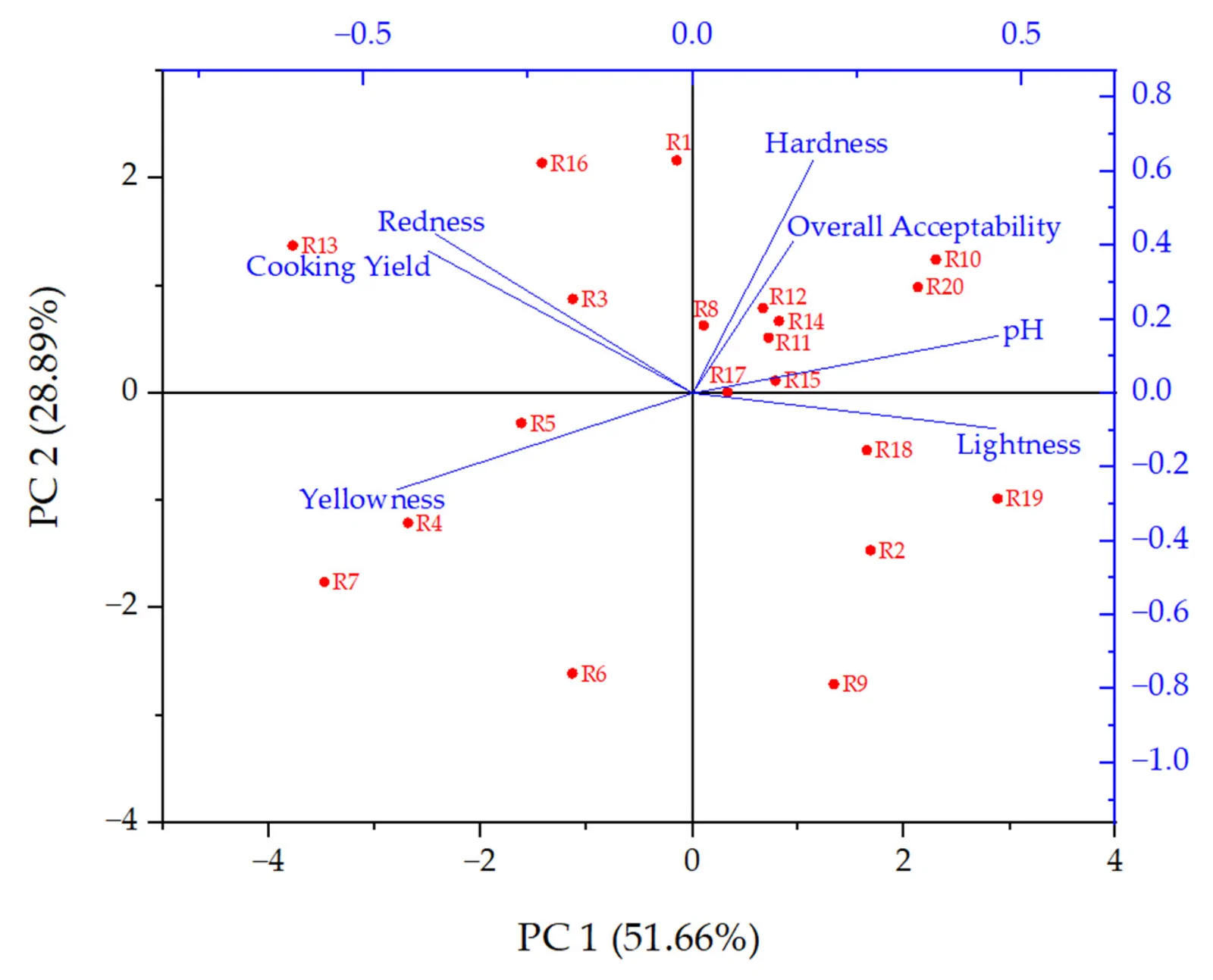
PCA biplot illustrating the distribution of experimental runs (R1–R20) and the loading directions of pH, cooking yield, lightness, redness, yellowness, hardness, and overall acceptability. PC1 and PC2 explain 51.66% and 28.89% of the total variance, respectively.

Principal component analysis was used to explore the multivariate relationships among the physicochemical, textural, and sensory responses of the 20 experimental formulations generated by the CCD (Figure 2). The RSM-derived optimized formulation was not included in the PCA; therefore, PCA was used only to explore the response structure, not to validate the RSM optimization. The first two principal components explained 80.55% of the total variance, with PC1 and PC2 accounting for 51.66% and 28.89%, respectively. PC1 primarily contrasted pH and lightness, which showed positive loadings, with cooking yield, redness, and yellowness, which showed negative loadings. PC2 was most strongly associated with hardness and overall acceptability, while redness and cooking yield also showed positive loadings on this component. Cooking yield and redness had similar loading directions, suggesting a positive association between these responses, whereas their loading directions contrasted with that of lightness. Similarly, pH and lightness showed comparable positive PC1 loadings, while yellowness was positioned in the opposite direction. These patterns represent exploratory associations among the measured responses and should not be interpreted as evidence of causal relationships.

**Figure 3:**
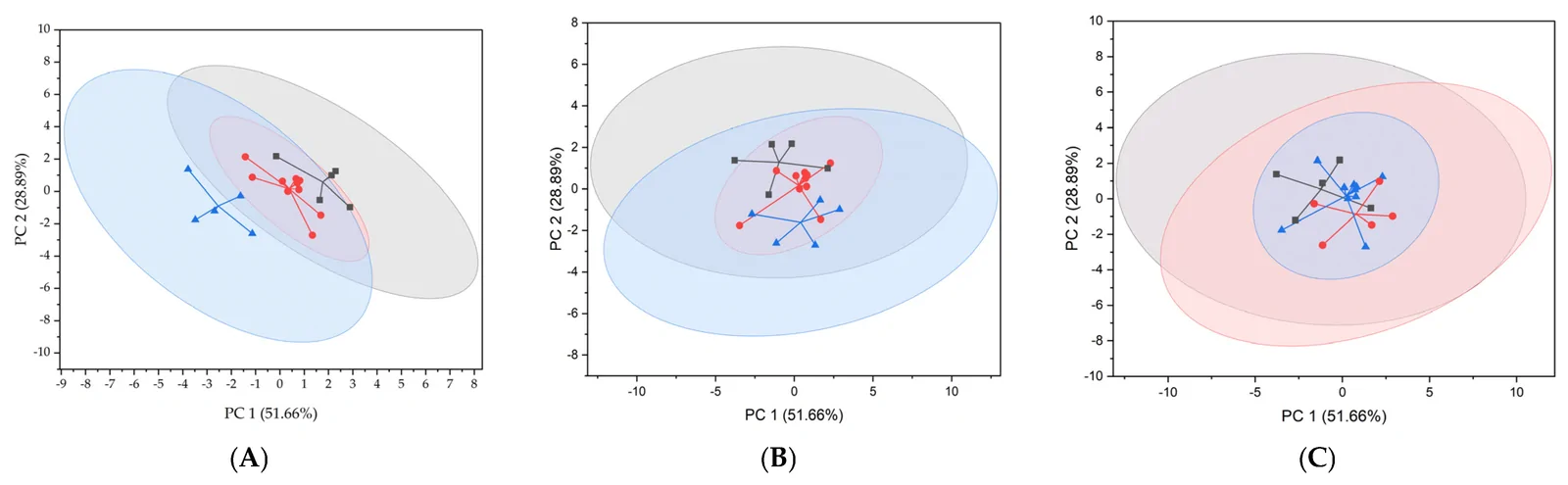
PCA score plots of the 20 CCD experimental runs grouped according to the levels of (**A**) apple pomace powder, (**B**) turkey skin, and (**C**) iced water. For each factor, coded levels <0, =0, and >0 were classified as low (n = 5), medium (n = 10), and high (n = 5), respectively. PC1 and PC2 explain 51.66% and 28.89% of the total variance, respectively. Black squares, red circles, and blue triangles indicate low, medium, and high factor levels, respectively. Ellipses indicate 95% confidence regions for each group.

The twenty CCD runs were also grouped by the coded levels of apple pomace powder (APP), turkey skin (TS), and iced water (IW) (Figure 3). For each factor, runs with coded levels below 0 were classified as low, those at coded level 0 as medium, and those above 0 as high. Accordingly, each factor comprised 5 low-level runs, 10 medium-level runs, and 5 high-level runs. In Figure 3A, grouping by APP level showed comparatively greater separation along PC1 than the groupings based on TS (Figure 3B) and IW (Figure 3C). Low-APP formulations tended to occur toward the positive side of PC1, corresponding to the loading directions of pH and lightness, whereas high-APP formulations tended toward the negative side of PC1, corresponding to the side of the PCA space associated with cooking yield, redness, and yellowness. Medium-APP formulations occupied an intermediate region, although overlap among the confidence ellipses remained evident. In contrast, the TS- and IW-based groupings showed moderate to substantial overlap in the PCA score space. These grouped score plots were used only to visualize multivariate patterns among the experimental formulations. The apparent differences in separation do not establish the magnitude or statistical significance of the effects of APP, TS, or IW. The effects and relative importance of the formulation factors were therefore evaluated from the RSM regression coefficients and ANOVA results rather than from visual inspection of the PCA plots.

## 4. Conclusions

In this study, we evaluated the formulation of cooked sausages using apple pomace powder and poultry-industry by-products to support food fortification. This study illustrates that response surface methodology is a useful tool for optimizing the formulation of cooked sausages and for exploring the relationships between independent and response variables. The results showed that apple pomace powder, turkey skin, and iced water had significant effects on most of the physicochemical, textural, and sensory properties of cooked sausages. The current study illustrates that the linear model was sufficient to describe and predict responses for pH, lightness (L*), redness (a*), overall acceptability, and the modified (quadratic) model for cooking yield, yellowness (b*), and hardness, respectively. The optimum condition was obtained through numerical optimization using the desirability function. The optimized formulation of cooked sausages was 4.45% apple pomace powder, 23.19% turkey skin, and 15.73% iced water. Experimental validation showed that the measured responses were generally consistent with the RSM predictions, supporting the suitability of the developed models for formulation optimization.

## Figures and Tables

**Table 1:** Independent variables and their corresponding coded and actual levels for optimizing cooked sausages.

Independent Variable	Symbol	Coded Levels				
		−α	−1	0	+1	+α
Apple pomace powder (%)	X_1_	−0.364 *	1	3	5	6.364
Turkey Skin (%)	X_2_	16.591	20	25	30	33.409
Iced Water (%)	X_3_	9.887	15	22.5	30	35.113

* The negative axial value for APP (−0.364%) indicates that the design space extends below zero, which was treated as 0% APP.

**Table 2:** Central composite design and observed response values for cooked sausage formulations.

Run	Independent Variables			Responses						
	Apple Pomace Powder (%)	Turkey Skin (%)	Iced Water (%)	pH	Cooking Yield	Lightness	Redness	Yellowness	Hardness	Overall Acceptability
1	1	20	15	5.8	87.36	76.15	4.54	9.83	57.8	8.1
2	3	25	35.113	5.71	84.71	77.77	2.07	7.39	42.8	8
3	3	25	9.887	5.73	87.32	75.83	4.88	9.84	54.7	5.7
4	5	30	15	5.59	86.89	73.34	4.05	12.9	44.5	5.5
5	5	20	30	5.55	86.2	75.17	4.03	12.07	46.8	7.8
6	5	30	30	5.58	84.69	75.59	3.03	13.58	40.2	6.5
7	6.364	25	22.5	5.52	86.55	73.14	4.09	16.15	42	6
8	3	25	22.5	5.79	85.99	74.01	3.8	7.42	52.1	7
9	3	33.409	22.5	5.81	84.28	77.87	2.17	10.69	41.2	6.1
10	−0.364 *	25	22.5	5.85	85.42	79.05	3.46	7.1	57.8	8.1
11	3	25	22.5	5.72	85.09	75.83	3.56	7.91	55.3	7.4
12	3	25	22.5	5.78	85.45	75.51	3.9	7.55	53.3	7.6
13	5	20	15	5.57	88.64	73.02	5.69	13.53	50.2	7.2
14	3	25	22.5	5.8	85	74.75	3.7	7.51	54.1	7.5
15	3	25	22.5	5.75	85.4	76.51	3.5	7.78	54.3	6.5
16	3	16.591	22.5	5.61	87.76	74.56	4.4	10.32	56.5	8.8
17	3	25	22.5	5.71	85.41	75.94	3.8	7.4	52.3	6.4
18	1	30	15	5.83	85.67	79.01	3.02	7.96	51.8	6
19	1	30	30	5.85	84.11	79.23	2.05	7.96	51.9	7.1
20	1	20	30	5.81	85.02	77.55	3.01	7.07	56.8	8.5

* The negative axial value for APP (−0.364%) indicates that the design space extends below zero, which was treated as 0% APP.

**Table 3:** Coefficients and ANOVA parameters of the cooked sausages for pH, cooking yield, color properties (L*, a*, b*), hardness, and overall acceptability.

pH							
	Coefficients	Sum of Squares	df	Mean Square	F-Value	Significance	Contribution %
Intercept (α0)	5.72	-	-	-	-	-	-
A-Apple Pomace Powder (α1)	−0.1139	0.1771	1	0.1771	88.16	***	92.05
B-Turkey Skin (α2)	0.0334	0.0152	1	0.0152	7.59	*	7.9
C-Iced water (α3)	−0.0025	0.0001	1	0.0001	0.0412	ns	0.05
Model	-	0.1924	3	0.0641	31.93	***	-
Lack of Fit	-	0.0251	11	0.0023	1.61	ns	-
R^2^				0.8569			
Adjusted R^2^				0.8300			
Predicted R^2^				0.7913			
Adequate precision				19.1093			
Cooking Yield							
Intercept (α0)	85.39	-	-	-	-	-	-
A-Apple Pomace Powder (α1)	0.4511	2.78	1	2.78	22.43	***	10.34
B-Turkey Skin (α2)	−0.8576	10.05	1	10.05	81.10	***	37.39
C-Iced water (α3)	−0.9467	12.24	1	12.24	98.82	***	45.54
A^2^ (α11)	0.2164	0.6746	1	0.6746	5.45	*	2.51
B^2^ (α22)	0.2287	0.7540	1	0.7540	6.09	*	2.81
C^2^ (α33)	0.2270	0.7424	1	0.7424	5.99	*	2.76
Model	-	26.88	6	4.48	36.16	***	-
Lack of Fit	-	1.00	8	0.1255	1.04	ns	-
R^2^				0.9435			
Adjusted R^2^				0.9174			
Predicted R^2^				0.8328			
Adequate precision				21.6646			
Lightness							
Intercept (α0)	75.99	-	-	-	-	-	-
A-Apple Pomace Powder (α1)	−1.81	44.89	1	44.89	64.20	***	75.05
B-Turkey Skin (α2)	0.7942	8.61	1	8.61	12.32	**	14.39
C-Iced water (α3)	0.6797	6.31	1	6.31	9.02	**	10.55
Model	-	59.81	3	19.94	28.52	***	-
Lack of Fit	-	7.12	11	0.6469	0.7944	ns	-
R^2^				0.8424			
Adjusted R^2^				0.8129			
Predicted R^2^				0.7709			
Adequate precision				17.5795			
Redness							
Intercept (α0)	3.64	-	-	-	-	-	-
A-Apple Pomace Powder (α1)	0.3837	2.01	1	2.01	27.22	***	13.44
B-Turkey Skin (α2)	−0.6495	5.76	1	5.76	78.02	***	38.5
C-Iced water (α3)	−0.7253	7.19	1	7.19	97.29	***	48.06
Model	-	14.96	3	4.99	67.51	***	-
Lack of Fit	-	1.06	11	0.0966	4.06	ns	-
R^2^				0.9268			
Adjusted R^2^				0.9131			
Predicted R^2^				0.8723			
Adequate precision				28.9389			
Yellowness							
Intercept (α0)	7.59	-	-	-	-	-	-
A-Apple Pomace Powder (α1)	2.52	87.05	1	87.05	679.22	***	62.92
B-Turkey Skin (α2)	0.0382	0.0200	1	0.0200	0.1558	ns	0.01
C-Iced water (α3)	−0.5609	4.30	1	4.30	33.53	***	3.11
AC (α13)	0.2475	0.4900	1	0.4900	3.82	ns	0.35
BC (α23)	0.6125	3.00	1	3.00	23.42	***	2.17
A^2^ (α11)	1.47	31.04	1	31.04	242.21	***	22.44
B^2^ (α22)	1.07	16.55	1	16.55	129.15	***	11.96
C^2^ (α33)	0.4035	2.35	1	2.35	18.31	**	1.70
Model	-	138.83	8	17.35	135.40	***	-
Lack of Fit	-	1.20	6	0.1997	4.73	ns	-
R^2^				0.9899			
Adjusted R^2^				0.9826			
Predicted R^2^				0.9516			
Adequate precision				39.3008			
Hardness							
Intercept (α0)	52.66	-	-	-	-	-	-
A-Apple Pomace Powder (α1)	−4.63	292.22	1	292.22	64.40	***	50.74
B-Turkey Skin (α2)	−3.58	175.32	1	175.32	38.64	***	30.44
C-Iced water (α3)	−2.10	59.95	1	59.95	13.21	**	10.41
B^2^ (α22)	−1.33	25.74	1	25.74	5.67	*	4.47
C^2^ (α33)	−1.37	27.12	1	27.12	5.98	*	4.71
Model		575.96	5	115.19	25.39	***	-
Lack of Fit	-	55.87	9	6.21	4.06	ns	-
R^2^				0.9007			
Adjusted R^2^				0.8652			
Predicted R^2^				0.7084			
Adequate precision				17.8085			
Overall Acceptability							
Intercept (α0)	7.09	-	-	-	-	-	-
A-Apple Pomace Powder (α1)	−0.4563	2.84	1	2.84	18.25	***	18.54
B-Turkey Skin (α2)	−0.8084	8.93	1	8.93	57.28	***	58.29
C-Iced water (α3)	0.5102	3.56	1	3.56	22.82	***	23.24
Model		15.32	3	5.11	32.78	***	-
Lack of Fit	-	1.14	11	0.1036	0.3828	ns	-
R^2^				0.8601			
Adjusted R^2^				0.8338			
Predicted R^2^				0.7986			
Adequate precision				20.1094			

ns not significant for *p* > 0.05; * significant for *p* ≤ 0.05; ** significant for *p* ≤ 0.01; *** significant for *p* ≤ 0.001.

**Table 4:** Regression equations of actual factors.

Responses	Equation
pH	+5.73 − 0.06X_1_ + 0.007X_2_ − 0.0003X_3_
Cooking Yield	+100.09 − 0.10X_1_ − 0.63X_2_ − 0.31X_3_ + 0.054X_1_^2^ + 0.01X_2_^2^ + 0.004X_3_^2^
Lightness	+72.70 − 0.91X_1_ + 0.16X_2_ + 0.10X_3_
Redness	+8.49 + 0.19X_1_ − 0.13X_2_ − 0.10X_3_
Yellowness	+49.32 − 1.317X_1_ − 2.5X_2_ − 0.86X_3_ + 0.02X_1_X_3_ + 0.02X_2_X_3_ + 0.37X_1_^2^ + 0.04X_2_^2^ + 0.01X_3_^2^
Hardness	+38.27 − 2.31X_1_ + 1.94X_2_ + 0.81X_3_ − 0.05X_2_^2^ − 0.02X_3_^2^
OAA	+10.29 − 0.23X_1_ − 0.16X_2_ + 0.07X_3_

**Table 5:** Optimized formulation and predicted and experimental response values under the optimized conditions.

Optimum Conditions		Actual Levels
Apple Pomace Powder (%)		4.45
Turkey Skin (%)		23.19
Iced Water (%)		15.73
Response	Predicted values	Experimental Values
pH	5.63	5.58 ± 0.04
Cooking Yield (%)	87.21	87.89 ± 0.27
Lightness	73.78	73.75 ± 0.45
Redness	4.81	4.90 ± 0.06
Yellowness	11.35	13.49 ± 0.99
Hardness (N)	51.21	53.36 ± 0.53
Overall Acceptability	6.59	6.97 ± 0.12

## Data Availability

The original contributions presented in the study are included in the article, further inquiries can be directed to the corresponding author.

## Abbreviations

The following abbreviations are used in this manuscript:

APP	Apple Pomace Powder
TS	Turkey Skin
IW	Iced Water
RSM	Response Surface Methodology
CCD	Central Composite Design
*L**	Lightness
*a**	Redness
*b**	Yellowness
TPA	Texture Profile Analysis
OAA	Overall Acceptability
PCA	Principal Component Analysis
